# Editorial: Novel mechanisms, imaging approaches, and management strategies for anthracycline-induced cardiotoxicity

**DOI:** 10.3389/fcvm.2022.1109078

**Published:** 2023-01-04

**Authors:** René R. Sevag Packard, Eric H. Yang

**Affiliations:** ^1^Division of Cardiology, Department of Medicine, David Geffen School of Medicine, University of California, Los Angeles, Los Angeles, CA, United States; ^2^Ronald Reagan University of California at Los Angeles Medical Center, Los Angeles, CA, United States; ^3^Veterans Affairs West Los Angeles Medical Center, Los Angeles, CA, United States; ^4^Department of Physiology, David Geffen School of Medicine, University of California, Los Angeles, Los Angeles, CA, United States; ^5^Jonsson Comprehensive Cancer Center, University of California, Los Angeles, Los Angeles, CA, United States; ^6^Molecular Biology Institute, University of California, Los Angeles, Los Angeles, CA, United States; ^7^California NanoSystems Institute, University of California, Los Angeles, Los Angeles, CA, United States; ^8^UCLA Cardio-Oncology Program, Division of Cardiology, Department of Medicine, David Geffen School of Medicine, University of California, Los Angeles, Los Angeles, CA, United States

**Keywords:** anthracycline, cardiotoxicity, cardiac imaging, exercise oncology, mechanisms and management of anthracycline associated cardiac injury

## 1. Introduction

Anthracyclines remain one of the essential chemotherapy class drugs due to their effective antineoplastic properties in multiple adult and pediatric cancers. Decades of observed cardiotoxicity—that predominantly manifest as cardiomyopathy and/or heart failure (1)—have made anthracyclines a “flagship” drug responsible for the rise of cardio-oncology. Much progress has been made in understanding the mechanisms and clinical presentations of anthracycline-induced cardiotoxicity (AIC), with their known risk of acute or delayed-onset cardiac injury ranging from cardiac fibrosis (2) to focal or global ventricular dysfunction (3), compounded by the negligible ability of the mammalian heart to regenerate, contrary to other species (4, 5). Yet, despite these efforts, accurately predicting, and effectively providing cardioprotection to patients who are vulnerable to AIC remain elusive. This has led to intense basic, translational, and clinical research over the past decades in an effort to dissect and deepen our understanding of AIC. As topic editors, we are grateful to the contributing authors for their expertise and manuscripts published in *Frontiers in Cardiovascular Medicine* comprised of the latest state-of-the-art reviews and clinical studies on novel mechanisms, imaging approaches, and management strategies of AIC.

## 2. Pathobiological mechanisms

Al-Otaibi et al. address the genetics of AIC, including genomic variants associated with AIC, stratified according to presumed pathophysiologic mechanisms. The authors analyze overlapping genes implicated in AIC and other types of cardiomyopathies: truncating variants in the titin gene (TTNtv), BCL2 associated athanogene 3 (BAG3), lamin A/C (LMNA), and Myosin Heavy Chain 7 (MYH7). They further develop 11 identified genes specific for AIC, including retinoic acid receptor-g (RARG), solute carrier family 28 member 3 (SLC28A3), and Rac family small GTPase 2 (RAC2). For translational and clinical applicability, the authors present a framework for interpreting genetic reports and potential applications to patient management.

Antoniak et al. explore less appreciated aspects of AIC: thrombosis, cardiac atrophy, and programmed cell death. In particular, the authors detail pro-thrombotic effects of anthracyclines on vascular cells, blood flow perturbation, platelet activation, release of tissue factor-bearing extracellular vesicles, and thrombus formation. They develop anthracycline induction of p53 expression, necessary for inactivation of mammalian target of rapamycin (mTOR), a serine-threonine kinase essential for protein synthesis. Furthermore, anthracyclines also induce expression of muscle RING finger1 (MuRF1), a striated muscle specific ubiquitin ligase and a key mediator of cardiac atrophy. Finally, the authors scrutinize programmed cardiomyocyte death pathways induced by AIC, including apoptosis, necroptosis, ferroptosis, and pyroptosis, illustrating the complex biology of anthracycline-mediated cardiac injury.

## 3. Imaging approaches

With the continued developments and improvements in advanced multimodality imaging, our series further explores applications of echocardiography, cardiac computed tomography (CT), cardiac magnetic resonance (CMR) imaging, and nuclear and molecular cardiology in AIC. Piveta et al. prospectively scrutinize echocardiographic metrics associated with subsequent cardiotoxicity in *n* = 51 patients with breast cancer treated with a chemotherapy regimen containing anthracyclines. Echocardiograms were evaluated at baseline, after 120 and 240 mg/m^2^ cumulative doses of doxorubicin, and 6- and 12-month after treatment completion. Among multiple 2-D and 3-D strategies analyzed by the authors, only changes in 3-D global area strain (GAS) were associated with a subsequent decrease in left ventricular ejection fraction (LVEF). 3-D GAS is a promising metric which assesses myocardial deformation mainly in the subendocardial layer (6) and will require further large-scale prospective evaluation.

Anthracyclines continue to be used for a variety of hematologic malignancies and certain solid tumors in the pediatric population, and delayed cardiotoxicity remains a topic of concern. This is particularly relevant due to an overall increase in childhood cancer survivors reaching adulthood. Niemelä et al. combined two Finnish cohorts of childhood cancer survivors (*n* = 90) that were exposed to anthracyclines and compared to healthy controls (*n* = 86) to evaluate left ventricular longitudinal strain detection of cardiac dysfunction in a cross-sectional manner. The authors indicate that longitudinal strain may be a more sensitive method than LVEF to detect cardiac dysfunction in pediatric patients.

Feher et al. provide a review of novel cardiac CT methods for the assessment of AIC. Cardiac CT has an increasing number of indications in cardiovascular medicine and cardio-oncology (7). Beyond the evaluation of coronary arteries and cardiac function, the authors develop new CT techniques including myocardial deformation, extracellular volume measurement, coronary vasoreactivity, determination of microvascular dysfunction, and even nanoparticle-based molecular imaging, highlighting their potential applicability in AIC.

Mabudian et al. dissect CMR imaging measures of left ventricular volumes and function applied to AIC. The authors also explore quantitation of left ventricular mass, perfusion imaging, and tissue characterization by T1/T2 mapping and late gadolinium enhancement. In particular, they highlight myocardial fibrosis characterization by methods utilizing gadolinium contrast, as well as those that don't (native T1) such as T1 and T2 mapping which measure longitudinal and transverse relaxation times, respectively.

Jong et al. explore nuclear imaging in AIC, from pathobiology to the identification of molecular targets. Indeed, nuclear imaging can map molecular processes perturbed in AIC in a specific manner using radioactively labeled probes. Targeted pathways that have been studied in nuclear medicine include metabolic dysfunction (glucose uptake, oxidative metabolism, fatty acid metabolism, mitochondrial membrane potential measurement, and determination of reactive oxygen species), cell death, sympathetic innervation, myocardial perfusion and blood flow measurement, and detection of cardiac fibrosis. The authors further provide a clinical framework for potential applications of nuclear molecular imaging in predicting and reclassifying cardiotoxicity in cancer patients undergoing anthracycline treatment, in a role complementary to that of echocardiography.

## 4. Management strategies

Kang et al. explore a less well-known but an emerging topic of interest of AIC management, namely exercise as a potential therapeutic modality. This strategy is complementary to current approaches seeking to mitigate the risk and consequences of AIC, including decreasing cumulative anthracycline doses, use of pegylated liposomes for chemotherapy delivery, and concomitant treatment with dexrazoxane. The authors review the potential of exercise therapy to stimulate biochemical and physiological responses leading to cardioprotective effects, including decreased cardiomyocyte apoptosis, improved endothelial and microvascular function, and decreased reactive species production. The authors also provide a valuable comparison of exercise guidelines, applicable to exercise cardio-oncology, between various societies.

Finally, Vuong et al. review current and potential novel therapeutics for AIC. The authors categorize their review of treatment strategies into three categories. First, preventive and early-stage therapies which beyond dexrazoxane include neurohormonal therapies such as b-blockers and aerobic exercise. Second, moderate to end-stage therapies which include cardiac resynchronization therapy, mechanical circulatory support systems for advanced ventricular dysfunction, and also orthotopic heart transplantation. Finally, the authors develop areas of ongoing research with potential future applicability, including mechanism-specific pharmacotherapies targeting apoptosis, oxidative stress, and inflammation, and also stem cell and gene therapy.

## 5. Conclusions

While the road to understanding the mechanistic underpinnings of AIC has been a long one, there continues to be exciting advances made from the bench to the bedside that improve our ability to identify, protect against, and/or treat AIC. This science has exponentially grown in pace and scale because of the rapidly rising multidisciplinary field of cardio-oncology. Our series hopes to highlight the many advances made in recent years to understand one of the oldest known forms of cancer treatment associated cardiotoxicity. As cancer survival continues to improve with overall advances in therapies, attenuating cardiotoxicity is of the utmost importance in order to ensure that patients do not suffer from the double-edged sword of anthracyclines with short- and long-term cardiac consequences that may lead to increased comorbidity and mortality.

## Author contributions

Both authors listed have made a substantial, direct, and intellectual contribution to the work and approved it for publication.
